# Supplementary material for: Most ornamental plants on sale in garden centres are unattractive to flower-visiting insects
Source: PeerJ. 2017 Mar 7;5:e3066. doi: 10.7717/peerj.3066 (PMC5344017; doi:10.7717/peerj.3066)
Supplement: Table S1 [file peerj-05-3066-s001.pdf]

Table S1. Comparison of plant variety attractiveness to flower-visiting insects in relation to other surveys, all conducted in East Sussex, UK.

	This study[1]	Garbuzov & Ratnieks (2014)[2]	Garbuzov et al. (2015)[1]
Origanum vulgare			
Rank, range	1 - 6 of 68 - 74	1 - 11 of 32	-
Rank, mean	4.0	6.0	3 of 79
Rank, mean percentile	6%	19%	4%
Insect count/m^2, range	7.8 - 46.9	2.8 - 7.2	-
Insect count/m^2, mean	20.2	5.0	11.2
Sample size	6	2	1
Dahlia , open-flowered			
Rank, range	2 - 42 of 68	10 - 19 of 32	11 - 39 of 79
Rank, mean	13.6	15.0	21.0
Rank, mean percentile	20%	47%	27%
Insect count/m^2, range	0.2 - 11.3	1.4 - 2.8	0.1 - 1.8
Insect count/m^2, mean	6.9	1.9	1.0
Sample size	7	4	4
Lavandula angustifolia			
Rank, range	5 - 42 of 59 - 69	18 - 28 of 32	n/a
Rank, mean	25.6	22.8	
Rank, mean percentile	40%	71%	
Insect count/m^2, range	0.0 - 10.5	0.3 - 1.3	
Insect count/m^2, mean	3.5	0.9	
Sample size	5	12	
Nepeta			
Rank, range	7 - 37 of 66 - 74	6 - 7 of 32	-
Rank, mean	17.3	6.5	23 of 79
Rank, mean percentile	25%	20%	29%
Insect count/m^2, range	2.7 - 23.0	3.2 - 3.2	-
Insect count/m^2, mean	11.1	3.2	0.6
Sample size	3	2	1
Pelargonium			
Rank, range	24 - 57 of 68 - 74	32 of 32	54 - 68 of 79
Rank, mean	51.5	32.0	64.5
Rank, mean percentile	73%	100%	82%
Insect count/m^2, range	0.0 - 11.1	0.0 - 0.1	0.00 - 0.02
Insect count/m^2, mean	1.9	0.06	0.0
Sample size	6	2	4

[1]Attractiveness estimated at or near peak of flowering period; [2]Attractiveness estimated over the whole flowering period
