# Supplementary material for: Most ornamental plants on sale in garden centres are unattractive to flower-visiting insects
Source: PeerJ. 2017 Mar 7;5:e3066. doi: 10.7717/peerj.3066 (PMC5344017; doi:10.7717/peerj.3066)
Supplement: Table S2 [file peerj-05-3066-s002.pdf]

Table S2. Plant varieties surveyed in the six garden centres, arranged in the decreasing order of attractiveness to flower-visiting insects

(a) Garden Pride

Plant variety	Type	RHS PfP	Label	Mean marjoram score	Mean insect count/m^2
Helenium 'The Bishop'	Multi-year	+		1.89	14.71
Origanum vulgare	Multi-year	+	n/a	1.00	7.78
Hebe 'Blue Star'	Multi-year	+		0.87	6.78
Salvia 'Mystic Spires'	Multi-year	+	+	0.56	4.38
Lavandula angustifolia 'Hidcote'	Multi-year	+	+	0.47	3.67
Helenium 'Tip Top'	Multi-year	+		0.34	2.67
Nepeta 'Purple Haze'	Multi-year			0.34	2.65
Thymus vulgaris 'Silver Posie'	Multi-year	+	+	0.34	2.65
Penstemon 'Phoenix Red'	Bedding	+	+	0.25	1.95
Salvia x jamensis 'Hot Lips'	Multi-year	+		0.23	1.77
Achillea 'Sunny Seduction'	Multi-year	+		0.22	1.70
Geranium 'Rozanne'	Multi-year	+	+	0.21	1.67
Scabiosa 'Barocca'	Multi-year			0.21	1.63
Cosmos atrosanguineus 'Chocolate'	Multi-year			0.15	1.18
Salvia x sylvestris 'Rose Queen'	Multi-year	+		0.15	1.13
Spiraea 'Double Play Gold'	Multi-year	+		0.13	1.04
Gaillardia x grandiflora 'Mesa Yellow'	Multi-year	+		0.13	0.99
Rehmannia 'Walberton's Magic Dragon'	Multi-year			0.13	0.98
Campanula 'Iridescent Bells'	Multi-year		+	0.11	0.85
Dahlia 'Dalina Maxi Sonara'	Bedding	+		0.11	0.85
Spiraea japonica 'Little Princess'	Multi-year	+		0.11	0.83
Nemesia 'Ice Pink'	Bedding			0.09	0.74
Anthemis tinctoria 'EC Buxton'	Multi-year	+		0.09	0.68
Osteospermum 'Cannington Roy'	Multi-year			0.09	0.68
Scabiosa atropurpurea 'Chile Black'	Bedding	+		0.09	0.68
Dianthus 'Twinkle Star'	Multi-year			0.07	0.54
Knautia macedonica 'Mars Midget'	Multi-year	+	+	0.07	0.52
Nemesia 'Amelie'	Bedding			0.06	0.48
Campanula cochlearifolia	Multi-year		+	0.06	0.46
Coreopsis 'Calypso'	Multi-year	+		0.05	0.42
Agapanthus 'Lavender Haze'	Multi-year			0.05	0.41
Gladiolus 'Alba'	Multi-year			0.05	0.41
Gladiolus 'Charming Beauty'	Multi-year			0.05	0.41
Salvia 'Clotted Cream'	Multi-year	+	+	0.05	0.41
Alstroemeria 'Inticancha Dark Purple'	Multi-year		+	0.04	0.34
Rosa 'Noatraum' (Flower Carpet)	Multi-year			0.03	0.27
Salvia 'Lavender Dilly Dilly'	Multi-year	+		0.03	0.27
Verbena 'Strawberry Kiss'	Multi-year			0.03	0.27
Campanula garganica 'Mrs Resholt'	Multi-year			0.03	0.24
Fuchsia 'Genii'	Multi-year	+		0.03	0.21
Diascia 'Maritana Bluebelle'	Multi-year			0.03	0.20
Dahlia 'Amazone'	Bedding	+	+	0.02	0.19
Rosa 'Diamond Anniversary'	Multi-year			0.02	0.19
Achillea 'Saucy Seduction'	Multi-year	+		0.02	0.17
Trachelospermum jasminoides 'Tripod'	Multi-year			0.02	0.14
Hydrangea macrophylla 'Soft Pink Salsa'	Multi-year			0.02	0.14
Verbena 'Homestead Purple'	Multi-year			0.02	0.14
Verbena 'La France'	Multi-year			0.02	0.14
Erigeron karvinskianus 'Kew Profusion'	Multi-year	+		0.01	0.10
Rosa 'Glowing Pink	Multi-year		+	0.01	0.10
Hydrangea macrophylla 'Blue Ballad'	Multi-year			0.01	0.07
Veronica spicata 'Royal Candles'	Multi-year			0.01	0.07
Dianthus 'Passion'	Multi-year			0.00	0.00
Digitalis purpurea 'Dalmatian White'	Multi-year	+		0.00	0.00
Fuchsia 'Lady Thumb'	Multi-year	+		0.00	0.00
Fuchsia 'Son of Thumb'	Multi-year	+		0.00	0.00
Fuchsia 'Tom Thumb'	Multi-year	+		0.00	0.00
Gaura lindheimeri 'Gaudi Pink'	Multi-year			0.00	0.00
Hydrangea macrophylla 'Pink Ball'	Multi-year			0.00	0.00
Hydrangea macrophylla 'Red Reggae'	Multi-year			0.00	0.00
Isotoma axillaris 'Fairy Carpet'	Multi-year			0.00	0.00
Knautia macedonica 'Thunder and Lightning'	Multi-year	+		0.00	0.00
Nemesia 'Nuvo Rose'	Bedding			0.00	0.00
Nemesia 'Nuvo White'	Bedding			0.00	0.00
Phygelius aequalis 'Trewidden Pink'	Multi-year			0.00	0.00
Phygelius x rectus 'Moonraker'	Multi-year			0.00	0.00
Verbena 'Edith Eddleman'	Multi-year			0.00	0.00
Veronica longifolia 'Charlotte'	Multi-year	+		0.00	0.00
Zantedeschia 'Captain Branco'	Multi-year			0.00	0.00

(b) Wyevale					
Plant variety	Type	RHS PfP	Label	Mean marjoram score	Mean insect count/m^2
Ageratum houstonianum 'High Tide Blue'	Bedding	+	+	2.47	14.92
Cosmos bipinnatus 'Sonata Carmine'	Bedding	+	+	2.34	14.15
Iberis 'Masterpiece'	Multi-year			1.32	7.96
Gypsophila muralis 'Teeny Deep Rose'	Bedding			1.25	7.54
Astilbe 'Younique Carmine'	Multi-year			1.21	7.31
Origanum vulgare	Multi-year	+	n/a	1.00	6.05
Veronica 'Inspire Pink'	Multi-year			0.84	5.09
Campanula 'Blue Moon'	Multi-year			0.73	4.41
Rosa 'Flower Carpet Pink' (Noatrawn)	Multi-year			0.63	3.83
Salvia nemorosa 'Caradonna'	Multi-year	+	+	0.63	3.82
Hebe 'Sparkling Sapphires'	Multi-year	+		0.62	3.77
Leucanthemum x superbum 'Lacrosse'	Multi-year	+	+	0.58	3.54
Rudbeckia hirta 'Toto Gold'	Bedding	+		0.47	2.83
Cosmos bipinnatus 'Sonata Pink'	Bedding	+		0.39	2.36
Lavandula stoechas 'Anouk'	Multi-year	+	+	0.38	2.31
Veronica longifolia 'First Lady'	Multi-year	+	+	0.37	2.21
Dianthus barbatus 'Dash Pink'	Multi-year	+		0.28	1.70
Dianthus barbatus 'Sweet Black Cherry'	Multi-year	+		0.28	1.68
Antirrhinum 'Peachy Bronze'	Bedding			0.25	1.51
Verbascum 'Lavender Lass'	Multi-year	+		0.21	1.27
Delosperma 'Jewel of Desert Moon Stone'	Multi-year			0.16	0.94
Gaura lindheimeri 'Gaudi Pink'	Multi-year			0.15	0.93
Sanvitalia 'Super Gold Upright'	Bedding			0.15	0.90
Petunia x hybrida 'Mystical Cha-Ching'	Bedding			0.15	0.88
Tagetes 'Antigua Vanilla'	Bedding			0.11	0.69
Salvia splendens 'Vista Purple'	Bedding	+		0.11	0.68
Fuchsia 'Beacon'	Multi-year	+		0.11	0.66
Bacopa 'Pink'	Bedding			0.10	0.58
Bacopa 'Blue'	Bedding			0.09	0.53
Salvia x jamensis 'Hot Lips'	Multi-year	+	+	0.08	0.48
Dianthus barbatus 'Purple Picotee'	Multi-year	+		0.08	0.48
Salvia farinacea 'Victoria Blue'	Bedding	+		0.08	0.47
Salvia farinacea 'Victoria Blue Bicolour'	Bedding	+		0.08	0.46
Lantana camara 'Lucky Sunrise Rose'	Bedding			0.08	0.46
Celosia argentea cristata 'Fresh Look Red'	Bedding			0.07	0.42
Petunia x hybrida 'Easy Wave Red Velour'	Bedding			0.06	0.39
Rosa 'Raspberry Royale'	Multi-year			0.06	0.34
Tagetes erecta 'Taishan Orange'	Bedding			0.05	0.29
Dahlia 'Pomponella Bicolor'	Bedding	+		0.05	0.28
Fuchsia 'Display'	Multi-year	+		0.04	0.27
Fuchsia 'Dollar Princess'	Multi-year	+		0.04	0.22
Dianthus caryophyllus nanus 'Lillipot Purple'	Bedding			0.03	0.19
Rehmannia 'Walberton's Magic Dragon'	Multi-year			0.03	0.17
Isotoma axillaris 'Avante-Garde Blue'	Multi-year			0.02	0.11
Alstroemeria 'Princess Letizia'	Multi-year			0.00	0.00
Angelonia angustifolia 'Archangel Blue'	Multi-year			0.00	0.00
Begonia x tuberhybrida 'Nonstop Scarlet Red'	Bedding			0.00	0.00
Celosia argentea cristata 'Fresh Look Orange'	Bedding			0.00	0.00
Dianthus barbatus 'Festival White Flame'	Multi-year	+		0.00	0.00
Dianthus caryophyllus nanus 'Lillipot Red'	Bedding			0.00	0.00
Diascia 'Flying Colours Red'	Multi-year			0.00	0.00
Fuchsia 'Tom Thumb'	Multi-year	+		0.00	0.00
Nicotiana x sanderae 'Cuba White'	Bedding			0.00	0.00
Nicotiana x sanderae 'Cuba Yellow'	Bedding			0.00	0.00
Pelargonium 'Calliope Deep Red'	Bedding			0.00	0.00
Pelargonium 'Calliope Lavender Rose'	Bedding			0.00	0.00
Pelargonium 'Calliope Rose Splash'	Bedding			0.00	0.00
Pelargonium peltatum 'Viletta Burgundy/White'	Bedding			0.00	0.00
Pelargonium peltatum 'Villetta Red'	Bedding			0.00	0.00
Petunia 'Pirouette Double Blue'	Bedding			0.00	0.00
Petunia 'Pirouette Double Purple'	Bedding			0.00	0.00
Petunia 'Satina Purple'	Bedding			0.00	0.00
Phygelius 'Funfare Coral'	Multi-year			0.00	0.00
Phygelius 'Funfare Wine'	Multi-year			0.00	0.00
Rosa 'Scarlet'	Multi-year			0.00	0.00
Rosa 'Sweet Dream'	Multi-year			0.00	0.00
Salvia splendens 'Vista Salmon'	Bedding	+		0.00	0.00
Tagetes erecta 'Vanilla'	Bedding			0.00	0.00
Tagetes patula 'Durango Bee'	Bedding	+		0.00	0.00
Verbena x hybrida 'Lanai Twister Blue'	Bedding	+		0.00	0.00

(c) Staverton

Plant variety	Type	RHS PfP	Label	Mean marjoram score	Mean insect count/m^2
Origanum vulgare	Multi-year	+	n/a	1.00	13.75
Dahlia 'Roxy'	Bedding	+		0.82	11.25
Dahlia 'Mystic Dreamer'	Bedding	+		0.77	10.61
Lavandula angustifolia 'Essence Purple'	Multi-year	+		0.77	10.53
Dahlia 'Bishop of Cantebury'	Bedding	+		0.67	9.23
Alstromeria 'Inticancha Dark Purple'	Multi-year			0.66	9.09
Dahlia 'Bishop of York'	Bedding	+		0.51	6.96
Agastache aurantiaca 'Apricot Sprite'	Bedding	+		0.49	6.79
Lavandula stoechas 'Puple Wings'	Multi-year	+		0.49	6.75
Hydrangea macrophylla 'Teller Red'	Multi-year			0.48	6.62
Penstemon 'Strawberries and Cream'	Multi-year	+		0.48	6.55
Salvia nemorosa 'Caradonna'	Multi-year	+		0.46	6.38
Hebe recurva 'Boughton Silver'	Multi-year	+		0.46	6.37
Agastache astromontana 'Pink Pop'	Multi-year	+		0.46	6.29
Dahlia 'Mystic Illusion'	Bedding	+		0.46	6.29
Leucanthemum x superbum 'Snow Lady'	Multi-year	+		0.43	5.94
Lavandula stoechas 'Snowman'	Multi-year	+		0.41	5.63
Alstromeria 'Inticancha Red'	Multi-year			0.37	5.13
Lavandula stoechas 'Lilac Wings'	Multi-year	+		0.37	5.05
Alstromeria 'Inticancha Navayo'	Multi-year			0.31	4.27
Dahlia 'Bishop of Llandaff'	Bedding	+		0.27	3.72
Alstromeria 'Inticancha Bryce'	Multi-year			0.20	2.72
Scabiosa columbaria 'Pink Mist'	Multi-year	+		0.19	2.68
Lavandula angustifolia 'Munstead'	Multi-year	+		0.18	2.53
Verbena bonariensis 'Lollipop'	Multi-year	+		0.17	2.36
Rudbeckia x Echinacea 'Summerina Brown'	Multi-year			0.16	2.26
Buddleja davidii 'Southcombe Splender'	Multi-year	+		0.16	2.21
Hydrangea macrophylla 'Teller Pink'	Multi-year			0.14	1.99
Clematis 'Angelique'	Multi-year			0.14	1.96
Agapanthus 'Blue Storm'	Multi-year			0.13	1.80
Rosa 'Conservation (Coldimple)'	Multi-year			0.13	1.77
Salvia nemorosa 'East Friesland'	Multi-year	+		0.13	1.75
Echinacea purpurea	Multi-year	+	+	0.12	1.70
Rosa 'Amber Sweet Dream (Fryritz)'	Multi-year			0.12	1.65
Hebe 'Addenda'	Multi-year	+		0.11	1.52
Erica x williamsii 'Ken Wilson'	Multi-year			0.11	1.52
Rosa 'Genesis (Fryjuicy)'	Multi-year			0.11	1.47
Clematis 'Diana's Delight'	Multi-year			0.10	1.33
Solanum rantonnetti 'Standard'	Bedding			0.10	1.33
Lavandula stoechas 'Lusi Purple'	Multi-year	+		0.10	1.32
Clematis 'Samaritan Jo'	Multi-year			0.09	1.18
Lavandula stoechas 'Lusi Pink'	Multi-year	+		0.08	1.16
Fuchsia 'Upright Alison Patricia'	Multi-year	+		0.08	1.16
Fuchsia 'Kath Wilson'	Multi-year	+		0.07	1.01
Erica mackayana 'Shining Light'	Multi-year			0.07	1.01
Rosa 'Queen Mother'	Multi-year			0.06	0.88
Achillea millefolium 'Desert Cream Deep Rose'	Multi-year	+	+	0.05	0.71
Fuchsia 'Sarah'	Multi-year	+		0.05	0.66
Fuchsia 'Albertina'	Multi-year	+		0.04	0.59
Fuchsia 'Carmel Blue'	Multi-year	+		0.04	0.59
Fuchsia 'General Monk'	Multi-year	+		0.04	0.59
Tiarella 'Pink Skyrocket'	Multi-year			0.04	0.52
Rosa 'Lavender Symphonies'	Multi-year			0.03	0.47
Fuchsia 'Display'	Multi-year	+		0.03	0.44
Verbena bonariensis	Multi-year	+	+	0.03	0.38
Rosa 'National Trust'	Multi-year			0.02	0.29
Roscoea x beesiana	Multi-year			0.02	0.26
Rosa 'Yellow Patio'	Multi-year			0.02	0.24
Fuchsia 'Upright Koralle Fulgens'	Multi-year	+		0.01	0.07
Achillea millefolium 'Desert Cream Cream'	Multi-year	+	+	0.00	0.00
Achillea millefolium 'Desert Cream Red'	Multi-year	+	+	0.00	0.00
Erica cinerea 'Golden Sport'	Multi-year	+		0.00	0.00
Erica cinerea 'Providence'	Multi-year	+		0.00	0.00
Geranium 'Rozanne'	Multi-year	+		0.00	0.00
Rosa 'Crimson Showers'	Multi-year			0.00	0.00
Rosa 'Remembrance (Harxampton)'	Multi-year			0.00	0.00
Rosa 'Sweet Dream (Frymincot)'	Multi-year			0.00	0.00
Viola 'Martin'	Multi-year			0.00	0.00
Viola cornuta 'Victoria's Blush'	Multi-year			0.00	0.00

(d) Marchants					
Plant variety	Type	RHS PfP	Label	Mean marjoram score	Mean insect count/m^2
Foeniculum vulgare 'Smokey'	Multi-year	+		1.65	47.08
Perovskia atriplicifolia 'Blue Spire'	Multi-year	+		1.28	36.67
Helenium 'Ruby Tuesday'	Multi-year	+		1.15	32.92
Helenium 'Lambada'	Multi-year	+		1.08	30.79
Helenium 'Flamendes Katchen'	Multi-year	+		1.01	28.97
Origanum vulgare	Multi-year	+	n/a	1.00	28.56
Helenium 'Zimbelstern'	Multi-year	+		0.89	25.46
Nepeta nuda	Multi-year			0.81	23.02
Sanguisorba tenuifolia 'Stand Up Comedian'	Multi-year			0.77	21.96
Helenium 'Kupferzwerg'	Multi-year	+		0.74	21.24
Helenium 'Fiesta'	Multi-year	+		0.72	20.48
Euphorbia donii 'Amjillasa'	Multi-year			0.71	20.16
Agastache rugosa	Multi-year	+		0.70	20.00
Sphaeralcea incana 'Sourup'	Multi-year			0.70	19.91
Veronicastrum virginicum 'Album'	Multi-year	+		0.65	18.52
Persicaria amplexicaulis 'Rosea'	Multi-year	+		0.63	17.94
Campanula lactiflora 'Prichard's Variety'	Multi-year	+		0.62	17.80
Helenium 'Die Blonde'	Multi-year	+		0.62	17.71
Helenium 'Rauchtopas'	Multi-year	+		0.62	17.71
Helenium 'Rubinzwerg'	Multi-year	+		0.48	13.78
Geranium 'Dilys'	Multi-year	+		0.46	13.05
Heuchera villosa 'Autumn Bride'	Multi-year			0.45	12.86
Polemonium archibaldiae	Multi-year			0.43	12.41
Persicaria amplexicaulis 'Jo & Guido's Form'	Multi-year	+		0.43	12.24
Pelargonium graveolens	Multi-year			0.39	11.11
Verbena hastata	Multi-year			0.39	11.11
Lythrum virgatum	Multi-year	+		0.38	10.82
Allium senescens 'Summer Beauty'	Multi-year	+		0.37	10.61
Persicaria amplexicaulis 'Taurus'	Multi-year	+		0.32	9.26
Veronicastrum virginicum 'Kobaltkaars'	Multi-year	+		0.29	8.33
Helianthus 'Carine'	Multi-year			0.28	7.94
Persicaria amplexicaulis 'Firedance'	Multi-year	+		0.28	7.91
Persicaria amplexicaulis 'Summer Dance'	Multi-year	+		0.27	7.83
Galega x hartlandii 'Alba'	Multi-year			0.27	7.72
Salvia verticillata 'Hannay's Blue'	Multi-year	+		0.25	7.23
Veronicastrum virginicum 'Incarnatum'	Multi-year	+		0.24	6.88
Alstroemeria 'Mars'	Multi-year			0.22	6.35
Calamintha nepeta	Multi-year	+		0.21	5.95
Gaura lindheimeri 'Pink Form'	Multi-year	+		0.20	5.73
Tulbaghia violacea 'Alba'	Bedding			0.17	4.76
Salvia x jamensis 'Silas Dyson'	Multi-year	+		0.16	4.63
Campanula lactiflora 'Dixter Presence'	Multi-year	+		0.16	4.46
Salvia 'Christine Yeo'	Multi-year	+		0.13	3.66
Sanguisorba canadensis (hybrid)	Multi-year			0.11	3.24
Salvia microphylla 'Cerro Potosi'	Multi-year			0.08	2.31
Phlox paniculata 'Uspekh'	Multi-year	+		0.08	2.27
Crocosmia 'Gerbe D'or'	Multi-year			0.08	2.22
Phlox paniculata 'Burgi'	Multi-year	+		0.08	2.22
Aconitum napellus 'Bergfurst'	Multi-year			0.07	1.96
Lythrum salicaria 'Blush'	Multi-year	+		0.06	1.82
Phlox paniculata 'Veg Plot White'	Multi-year	+		0.06	1.77
Agapanthus 'Best Barn Blue'	Multi-year			0.05	1.52
Persicaria amplexicaulis 'Alba'	Multi-year	+		0.05	1.46
Anaphalis yedoensis	Multi-year			0.05	1.46
Lythrum virgatum 'Rose Queen'	Multi-year	+		0.05	1.41
Crocosmia 'Severn Sunrise'	Multi-year			0.05	1.33
Crocosmia 'Okavango'	Multi-year			0.04	1.26
Phlox paniculata 'Marchants Darkest'	Multi-year	+		0.04	1.08
Verbena bonariensis	Multi-year	+		0.04	1.06
Helenium 'Sahin's Early Flowerer'	Multi-year	+		0.04	1.04
Agapanthus 'Marchant's Best Blues'	Multi-year			0.04	1.01
Achillea 'Mondpagode'	Multi-year	+		0.03	0.80
Tulbaghia violacea 'Harry Hay'	Multi-year			0.03	0.79
Phlox paniculata 'Konigin Der Nacht'	Multi-year	+		0.02	0.66
Kalimeris incisa	Multi-year			0.02	0.58
Lobelia x speciosa 'Dark Crusader'	Multi-year			0.01	0.19
Alstroemeria 'Red Elf'	Multi-year			0.00	0.00
Diascia 'Emma'	Multi-year			0.00	0.00
Eucomis comosa 'Sparkling Burgundy'	Multi-year			0.00	0.00
Phlox paniculata 'David'	Multi-year	+		0.00	0.00
Phlox paniculata 'Monica Lynden-Bell'	Multi-year	+		0.00	0.00
Phlox paniculata 'Rich Pink'	Multi-year	+		0.00	0.00
Phlox paniculata 'Veg Plot Pink'	Multi-year	+		0.00	0.00
Salvia microphylla 'Pink Blush'	Multi-year	+		0.00	0.00
Tulbaghia 'Marchant's Large Flowered'	Multi-year			0.00	0.00

(e) Hillier					
Plant variety	Type	RHS PfP	Label	Mean marjoram score	Mean insect count/m^2
Hebe 'Groen White'	Multi-year	+		2.86	51.52
Caryopteris x clandonensis 'Heavenly Blue'	Multi-year	+		2.05	36.90
Hebe 'Groen Pink'	Multi-year	+		1.74	31.31
Aster ageratoides 'Asmo'	Multi-year	+		1.53	27.54
Origanum vulgare	Multi-year	+	n/a	1.00	18.04
Hebe 'Addenda Donna Britta'	Multi-year	+		0.99	17.94
Erysimum 'Sugar Rush Yellow'	Multi-year	+	+	0.94	16.98
Tradescantia 'Blue and Gold'	Multi-year			0.92	16.67
Aster ageratoides 'Asran'	Multi-year	+		0.88	15.93
Buddleja davidii 'Buzz Sky Blue'	Multi-year	+	+	0.62	11.11
Viburnum tinus 'Gwenllian'	Multi-year	+		0.59	10.61
Aster novi-belgii 'Starletta Pink'	Multi-year	+		0.58	10.53
Aster ageratoides 'Ashvi'	Multi-year	+		0.56	10.17
Penstemon 'Pensham Amelia Jayne'	Bedding	+		0.47	8.49
Erigeron karvinskianus 'Stallone'	Multi-year	+	+	0.47	8.49
Gaillardia x grandiflora 'Mesa Yellow'	Multi-year	+	+	0.43	7.74
Buddleja davidii 'Buzz Lavender'	Multi-year	+	+	0.39	7.07
Geranium 'Mavis Simpson'	Multi-year	+	+	0.37	6.60
Caryopteris x clandonensis 'Hint of Gold'	Multi-year	+		0.35	6.23
Penstemon 'Pensham Plum Jerkum'	Bedding	+		0.31	5.66
Erysimum 'Sugar Rush Bronze'	Multi-year	+	+	0.31	5.56
Erysimum 'Sugar Rush Primrose'	Multi-year	+	+	0.29	5.21
Rosa 'Flower Carpet White' (Noaschnee)	Multi-year			0.27	4.94
Campanula poscharskyana 'Addenda Adansa Pu	Multi-year	+		0.25	4.42
Rosa 'Iceberg' (Korbin)	Multi-year			0.24	4.33
Buddleja davidii 'Buzz Ivory'	Multi-year	+	+	0.22	3.89
Weigela 'Kosteriana Variegata'	Multi-year			0.20	3.61
Erysimum 'Sugar Rush Red'	Multi-year	+	+	0.19	3.47
Aubrieta 'Doctor Mules Variegata'	Multi-year	+		0.19	3.45
Ceratostigma willmottianum	Multi-year			0.19	3.40
Scabiosa 'Vivid Violet'	Multi-year		+	0.18	3.33
Echinacea purpurea 'PowWow White'	Multi-year	+	+	0.18	3.17
Salvia nemorosa 'Sensation Deep Rose'	Multi-year	+	+	0.18	3.17
Rosa 'Winchester Cathedral' (Auscat)	Multi-year			0.16	2.96
Crocosmia x crocosmiiflora 'Carmin Brilliant'	Multi-year			0.15	2.78
Aster novi-belgii 'Starletta White'	Multi-year	+		0.15	2.65
Verbena bonariensis	Multi-year	+	+	0.11	1.99
Calluna vulgaris 'Silver Fox'	Multi-year	+		0.10	1.85
Erica x watsonii 'Pink Pacific'	Multi-year		+	0.10	1.85
Hydrangea paniculata 'Grandiflora'	Multi-year	+		0.06	1.17
Crocosmia x crocosmiiflora 'Buttercup'	Multi-year			0.06	1.04
Crocosmia x crocosmiiflora 'George Davison'	Multi-year			0.05	0.98
Lavandula angustifolia 'Hidcote'	Multi-year	+		0.03	0.58
Calluna vulgaris 'Wickwar Flame'	Multi-year	+	+	0.03	0.51
Hydrangea paniculata 'Limelight'	Multi-year	+		0.02	0.35
Viola 'Pure White'	Bedding			0.02	0.30
Cyclamen hederifolium 'AmazeMe Pink'	Multi-year			0.01	0.16
Aster novi-belgii 'Mystery Lady'	Multi-year	+		0.00	0.00
Calluna vulgaris 'Firefly'	Multi-year	+	+	0.00	0.00
Chaenorhinum origanifolium 'Blue Dreams'	Multi-year			0.00	0.00
Cyclamen hederifolium 'AmazeMe White'	Multi-year			0.00	0.00
Dianthus 'Sunflor Cosmos kgr'	Multi-year			0.00	0.00
Dianthus 'Sunflor Paseo'	Multi-year			0.00	0.00
Viola 'Blue Blotch'	Bedding			0.00	0.00
Viola 'Copperfield'	Bedding			0.00	0.00
Viola 'Honeybee'	Bedding			0.00	0.00
Viola 'Rocky Tangerine'	Bedding			0.00	0.00
Viola 'True Blue'	Bedding			0.00	0.00
Viola cornuta 'Sorbet XP Delft Blue'	Bedding			0.00	0.00
Viola x wittrockiana 'Deltini Rose Pink'	Bedding			0.00	0.00

(f) Wych Cross

Plant variety	Type	RHS PfP	Label	Mean marjoram score	Mean insect count/m^2
Campanula poscharskyana 'Blue Waterfall'	Multi-year	+		3.11	145.83
Helenium 'Kanaria'	Multi-year	+		1.66	77.81
Achillea millefolium 'Lilac Beauty'	Multi-year	+		1.21	56.59
Origanum vulgare	Multi-year	+	n/a	1.00	46.91
Sedum spectabile 'Autumn Joy'	Multi-year	+		0.91	42.79
Clematis 'Cezanne'	Multi-year			0.86	40.42
Sedum spectabile 'Stardust'	Multi-year	+		0.84	39.30
Euphorbia x martini 'Baby Charm'	Multi-year			0.75	35.11
Sedum telephium 'Munstead Red'	Multi-year	+	+	0.67	31.24
Solidago sphacelata 'Golden Fleece'	Multi-year	+		0.65	30.56
Astrantia major 'Star of Beauty'	Multi-year	+		0.48	22.47
Caryopteris x clanodensis 'Heavenly Blue'	Multi-year	+		0.44	20.83
Rudbeckia triloba 'Prairie Glow'	Multi-year	+		0.44	20.45
Potentilla fruticosa 'Goldfinger'	Multi-year	+	+	0.43	20.30
Astrantia major 'Florence'	Multi-year	+	+	0.43	20.14
Penstemon 'Pensham Laura'	Bedding	+	+	0.42	19.89
Salvia x sylvestris 'Blauhugel'	Multi-year	+	+	0.40	18.67
Gaura lindheimeri 'Rosy Jane'	Multi-year	+	+	0.40	18.57
Astrantia major 'Buckland'	Multi-year	+	+	0.38	17.68
Calluna vulgaris 'Darkness'	Multi-year	+		0.37	17.46
Kniphofia 'Mango Popsicle'	Multi-year			0.35	16.50
Agastache foeniculum x rugosa 'Blue Fortune'	Multi-year	+	+	0.32	15.16
Persicaria amplexicaulis 'Golden Arrow'	Multi-year	+	+	0.32	14.80
Chelone obliqua	Multi-year			0.31	14.40
Liatris spicata 'Alba'	Multi-year	+		0.30	14.21
Helenium 'Salsa'	Multi-year	+		0.30	13.86
Persicaria bistorta 'Pink Elephant'	Multi-year	+		0.29	13.75
Potentilla fruticosa 'King Cup'	Multi-year	+		0.29	13.74
Lobelia x speciosa 'Sparkling Ruby'	Multi-year			0.27	12.73
Caryopteris x clanodensis 'Summer Sorbet'	Multi-year	+		0.26	12.41
Clematis texensis 'Princess Diana'	Multi-year			0.25	11.79
Kniphofia 'Pineapple Popsicle'	Multi-year			0.25	11.79
Clematis 'Corinne'	Multi-year			0.24	11.32
Viburnum tinus 'Eve Prince'	Multi-year	+		0.18	8.49
Potentilla fruticosa 'Medicine Wheel'	Multi-year	+		0.18	8.47
Clematis 'Diana's Delight'	Multi-year			0.18	8.25
Nemesia 'Ice Pink'	Bedding			0.17	7.86
Nepeta x faassenii	Multi-year	+	+	0.16	7.47
Sidalcea malviflora 'Elsie Heugh'	Multi-year	+	+	0.16	7.43
Scabiosa atropurpurea 'Butterfly Blue'	Bedding		+	0.16	7.37
Salvia uliginosa	Multi-year	+		0.14	6.76
Ceratostigma willmottianum 'Forest Blue'	Multi-year			0.13	6.32
Heuchera 'Paris'	Multi-year			0.13	5.99
Gentiana sino-ornata	Multi-year			0.11	5.21
Penstemon 'Pensham Loganberry Ice'	Bedding	+		0.11	5.16
Cyclamen hederifolium 'Pink'	Multi-year			0.10	4.55
Abelia x grandiflora 'Lucky Lots'	Multi-year			0.09	4.27
Cosmos atrosanguineus 'Chocolate'	Bedding			0.09	4.13
Rosa 'Ballerina'	Multi-year			0.08	3.77
Achillea millefolium 'Tutti Frutti Pomegranate'	Multi-year	+		0.08	3.68
Rosa 'Marjorie Fair' (Harhero)	Multi-year			0.07	3.48
Crocosmia 'George Davidson'	Multi-year			0.06	3.03
Fuchsia 'Royal Academy'	Multi-year	+		0.06	2.97
Silene dioica 'Rollie's Favourite'	Multi-year			0.06	2.95
Fuchsia 'Tom Thumb'	Multi-year	+	+	0.05	2.40
Fuchsia 'Lady Thumb'	Multi-year	+	+	0.05	2.38
Fuchsia 'Remembrance'	Multi-year	+		0.05	2.32
Rosa 'Coral Flower Carpet' (Noala)	Multi-year			0.04	1.95
Verbena bonariensis 'Lollipop'	Multi-year	+		0.03	1.60
Salvia x jamensis 'Hot Lips'	Multi-year	+		0.03	1.55
Cyclamen hederifolium 'White'	Multi-year			0.03	1.21
Rosa 'Bonica' (Meidomonac)	Multi-year			0.02	0.97
Fuchsia 'Genii'	Multi-year	+	+	0.01	0.66
Rosa 'Lavender Lassie'	Multi-year			0.01	0.65
Geranium 'Rozanne'	Multi-year	+	+	0.01	0.57
Lobelia x speciosa 'Starship Scarlet'	Multi-year			0.00	0.00
Rosa 'Grace' (Auskeppy)	Multi-year			0.00	0.00
